# Survey of policy for MRSA screening in English cataract surgical units and changes to practice after updated National guidelines

**DOI:** 10.1186/1471-2415-13-80

**Published:** 2013-12-17

**Authors:** Lavnish Joshi, Stephanie K West, Luke Herbert

**Affiliations:** 1 Department of Clinical Ophthalmology, Institute of Ophthalmology, University College London, 11-43 Bath Street, London EC1V 9EL, UK; 2 Bristol Eye Hospital, Lower Maudlin Street, Bristol, UK; 3 East Surrey Hospital, Surrey & Sussex Healthcare Trust, Redhill, Surrey, UK

**Keywords:** Healthcare surveys, Meticillin-resistant Staphylococcus aureus, Phacoemulsification, Screening

## Abstract

**Background:**

National guidelines on MRSA (methicillin-resistant *Staphylococcus aureus*) screening policy in England have changed on a number of occasions, but there is limited data on its influence at a local level. The aim of this study was to determine if changes in National policy influenced preoperative screening of cataract patients for MRSA.

**Methods:**

A structured telephone survey was conducted on all 133 ophthalmology units in England in 2004 and again in 2007 for the initial responders, after a change in national policy.

**Results:**

A total of 74 units (56%) responded in 2004 and 71 units (96% of initial respondents) in 2007. In 2004, 57% of units screened for MRSA. They screened groups at high risk of carriage, including patients with previous MRSA (93%) and patients from Nursing homes (21%). Swab sites included the nose (100%), eyes (31%) and perineum (62%). In 2007, there was no significant change in the number of units that screened for MRSA (57% vs 66%; p = 0.118; McNemar test). However, more units screened for MRSA in patients from nursing/residential homes (21% vs 51%; *p* = 0.004, McNemar test), and in patients who had recent admission to hospital (12% vs 36%; *p* = 0.003). In the second survey, 3 units (6%) now screened patients who were close relatives of MRSA carriers.

**Conclusion:**

This survey has highlighted inconsistences in MRSA screening practice of day-case cataract surgery patients across England after 2 major national policy changes. A change in DoH policy only led to more units screening patients for MRSA from high risk groups.

## Background

In the UK, hospital acquired infections (HAIs) are estimated to affect 9% of inpatients, with a cost of £1 billion per annum to the National Health Service (NHS) [1]. Twenty percent (20%) of HAIs are caused by methicillin-resistant *Staphylococus aureus* (MRSA). Since the mid-1990s, this so-called ‘hospital superbug’ was a topic of media and political concern in the UK [2].

Revised guidelines published in 1998 addressed MRSA control in English hospitals through a more flexible ‘targeted’ approach rather than the initial ‘search and destroy’ principle [3]. Patients were stratified according to the level of perceived risk to them from MRSA and a range of management options were described for each level of risk. According to these guidelines, elective cataract surgery was considered as low risk, thus preoperative screening was not necessary.

The increasing politicisation of MRSA and a review of our preoperative assessment procedures for patients undergoing cataract surgery prompted us to question whether MRSA screening should take place. We conducted a questionnaire to ascertain the practice of ophthalmology departments across England in 2004. Another survey of the initial respondents was carried out again in 2007 to see if the policy in these departments had changed since the publication of new the UK MRSA guidance [4] and the England Department of Health (DoH) document on MRSA screening [5].

## Methods

A structured telephone questionnaire (Table 1) was conducted for all National Health Service (NHS) ophthalmology units in England listed in the Royal college of Ophthalmologists’ Directory of Training Posts 2003–2004 [6]. A total of 133 units were identified. This was initially carried out in March-June 2004 and again, for all initial respondents, in May-July 2007 to determine if changes in practice had occurred. Our point of contact in each unit was a member of staff undertaking the pre-assessment of cataract patients. At least 3 attempts were made to contact staff.

**Table 1:** Structured telephone questionnaire

**Number**	**Question**
1	Does your department routinely screen any preoperative cataract patients for MRSA?
2	Which groups of patients do you screen?
3	Which sites do you take a swab from?
4	If patients are found to be positive, who treats them?
5	Have you ever had a case of MRSA endophthalmitis?

This research did not involve human data or material, hence ethical approval was not sought for this study.

### Quantitative analysis- statistical tests

The McNemar’s test for categorical dichotomous (Yes/No answers) paired data was used to compare questions in 2004 vs 2007 and determine if any significant change in policy on MRSA screening had occurred. 2 × 2 contingency tables were constructed for each of the questions asked above.

### Qualitative analysis

Free text comments made by respondents were documented; common themes were identified and coded (based on basis of screening practice) before data analysis by one of the authors (LJ).

## Results

### Initial survey

#### Response rate

In the initial 2004 survey, 74 (56%) units responded out of a total of 133 in England (Table 2). The units that did not respond included those that felt the survey was too sensitive in nature (they were concerned about risk information becoming available to the media) whereas other units could not be contacted despite several attempts.

**Table 2:** MRSA screening practices for preoperative cataract surgery patients in English units in 2004 and 2007

	**2004**		**2007**	
	**Total**	**%**	**Total**	**%**
Response				
Yes	74	56	71	96
No	133	44	3	4
Screen for MRSA				
Yes	42	57	47	66
No	32	43	24	34
Groups screened for MRSA				
Nursing/Residential Home*	9	21	24	51
Recent Hospital Admissions*	5	12	17	36
Previously MRSA + ve	39	93	38	81
Overnight Stay	4	10	4	9
From Hospitals abroad	5	12	6	13
Other Hospitals	8	19	8	17
Wounds/Indwelling catheters	9	21	14	30
Close relatives of MRSA carriers	0	0	3	6
Diabetics	0	0	1	2
Sample Sites for Swab				
Don’t Know	1	2.5	2	4
GP informed	2	5	1	2
Nose	39	100	43	98
Eye	12	31	8	18
Throat	15	38	18	41
Axilla	11	28	12	27
Groin/Perineum	24	62	23	52
Wounds/Catheters	9	23	14	32
1 site	6	15	5	11
More than 1 site	33	85	39	89
Treatment Responsibility				
Don’t Treat- Placed Last on List	6	14	0	0
GP*	31	74	42	93
Infection Control	5	12	6	13
Eye Nurse	3	7	4	9
Combination	6	14	7	16
Case of MRSA endophthalmitis	4/74		2/71	

Survey in 2007 was repeated with initial responders only.

*Indicates that there was a significant difference between the paired responses in 2004 vs 2007 (Mc Nemar test).

### Units that screened for MRSA in high risk groups

43% of units screened no pre-operative cataract patients for MRSA. The units that did screen for MRSA (57%), screened groups at high risk of carriage, including patients with previous MRSA (93%), patients from nursing homes (21%), patients from other hospitals (21%), recent history of admission for any reason (12%; range varied 6–18 months) and patients from abroad (12%).

### Screening method-sample site

If a patient did require screening, 2 units would inform the General Practioner (GP or primary care physician) to make necessary arrangements for swabs but the unit would not swab the patient themselves. 1 unit was not certain what sites were required to be sampled. For the other 39 units, swab sites included the nose (100%), groin (62%), throat (38%), axilla (28%), wounds/catheters (23%) and the eye (31%). 15% of units swabbed from a single site only.

### Responsibility for treatment (eradication therapy)

After screening, if MRSA was identified, 6 units (14%) would not treat patients but on the day of the admission, isolate them and place them at the end of the list. Of those that treated, a number of health professionals were responsible for the treatment of MRSA colonisation (eradication therapy). GPs were asked to treat in 31 units (79%), a member of the eye team treated patients in 3 units (7%) and the infection control team treated them in 5 units (7%).

### Experience of MRSA endophthalmitis

4 units (5%) were aware of at least one case of MRSA endophthalmitis. Out of these, 1 unit did not screen for MRSA.

### Comparison with second survey

#### Response rate

The second telephone survey in 2007, involved the 74 responders to the initial survey. Contact with 71 units was successful (96%). Two units refused to answer any questions due to the sensitive nature (and 1 unit could not be contacted.

#### High risk groups that were screened

In 2007, 47 units (66%) screened high-risk preoperative patients for MRSA compared with 42 (57%) units in 2004. 10 additional units started to screen for MRSA in high risk groups, however, 3 units stopped screening any patients for it. For the paired responses, there was no significant change in the decision to screen for MRSA between 2004 and 2007 (*p* = 0.118; *n* = 37, McNemar test).

More units screened for MRSA in patients from nursing/residential homes (51% vs 21%) and this was a significant change for the paired responses (*p* = 0.004, *n* = 37, McNemar test). Screening of recent hospital admissions also increased (36% vs 12%), which was also a significant change (*p* = 0.003; *n* = 37, McNemar test). In the second survey, 3 units (6%) now screened a new high risk group - patients who were close relatives of MRSA carriers.

#### Screening method-sample site

The nose was still the primary swab site for 98% of units, however 1 unit now swabbed wounds only for MRSA. There was no significant change in sample sites for the paired responses (*p* > 0.10; McNemar test).

#### Treatment responsibility

In 2007, more units stated that the responsibility for MRSA eradication therapy was with the GP (93% vs 74%). The source of these new responses were from the units that had started to screen in 2007, so there was no significant change in the paired responses (*p* = 0.18; McNemar test). In 2004, 5 out of the 6 units that did screen, but did not treat positive patients (instead preferring to place them at the end of the list), now treated these patients in 2007.

#### Other comments by respondents

Other comments by the respondents have been summarised in Table 3.

**Table 3:** Selected comments by respondents

	**Selected comments**
1	Reasons for not screening:
	”microbiology says it costs too much”, “swabs were not being followed up and GP doesn’t always treat”, “we rely on swabs from previous admissions, which if positive, result in a patient being placed at the end of the operating list”
2	The reason given for not screening anymore in 2007:
	”we had a new policy given by infection control”
3	The reason given why policy changed to start screening in 2007:
	”new infection control policy to screen all elective surgeries. We did this for 3 months but microbiology then stated that for cataract surgery it should be limited to high risk patients”
4	Unit that did not screen for MRSA, despite having a case of MRSA endophthalmitis:
	”it was a community-acquired strain”
5	Change in swab sites:
	”swabbing the groin was no longer appropriate in an eye clinic, and were told the nose is sufficient now”

## Discussion

The results of this survey highlight the inconsistencies in the policy of eye departments in England for the pre-operative screening for MRSA in patients undergoing cataract surgery. It seems that either some units are using resources to unnecessarily screen and treat patients, or other units are putting patients at unnecessary risk of MRSA infection by not screening. A postal survey has been conducted previously in 2005 [7], prior to the recent DoH (England) and UK guidelines, which demonstrated a similar percentage (67%) of UK eye departments screening for MRSA prior to cataract surgery. Our survey extends these findings by determining if there was any significant change in practice before and after a change in DoH guidelines.

When the initial survey was carried out in 2004, the guidance available for MRSA screening in the hospital setting was based on those suggested by the combined Working Party [3]. These offered flexibility by allowing individual hospitals to interpret these guidelines in the context of the local situation. However, it was not clear what the implications were for preoperative screening for patients undergoing cataract surgery.

When the second survey was conducted in 2007 a number of developments had occurred. This included the UK MRSA guidance [4] and DoH (England) document on screening for MRSA colonisation [5]. These guidelines stated that certain high risk groups should be screened routinely, but the guidelines acknowledged the importance of local infection control teams in deciding who should be screened. The definition of high risk patients depended on the MRSA risk status of the unit, the reason for admission (acute vs elective) and the likelihood of the patient being an MRSA carrier. Thus, elective cataract surgery patients would be classified as low risk.

Nevertheless, patients at high risk of carriage may have to be screened. The UK guidelines are explicit in recommending this [4]: “All patients who are at high risk for carriage of MRSA should be screened at the time of admission, unless .... being admitted to isolation facilities *and* it is not planned to attempt to clear them of MRSA carriage”. However, the DoH (England) document [5] is less prescriptive and suggests that “trusts should consider how local risk assessment can be done and screening implemented for these patient groups”. In our survey, although there was no significant change in the policy to screen for MRSA (57% vs 66%), the recent guidelines may have influenced some of the significant changes in practice that occurred when the second survey was carried out in 2007. In 2007, our survey of the initial respondents from 2004 demonstrated that more units were screening for MRSA in patients with a high risk of carriage (ie patients from nursing/residential homes, recent hospital admissions or close relatives of MRSA carriers). The practice to screen patients with a history of MRSA colonisation/infection remained the same during this period.

The variability between units regarding which groups to screen for MRSA may also be explained by the DoH (England) guidelines since they acknowledge the importance of local infection control teams in deciding who should be screened [5]. Thus, the decision by units not screen for MRSA, even in patients with high risk for carriage, may have been based on a sound local risk assessment.

So should patients undergoing cataract surgery, who are at high risk for carriage, be screened for MRSA? A risk assessment on MRSA carriers may be based on two concerns: the incidence and impact of an MRSA infection (ie endophthalmitis) and the risk of transmitting MRSA to others. To our knowledge, there is no reported evidence that MRSA MRSA carriage increases the risk of endophthalmitis. The incidence of MRSA endophthalmitis seems to vary. In a case series of 64 consecutive cases of endophthalmitis, 18.2% (total of 6) of isolates were identified as MRSA [8]. Another case series in the USA, demonstrated that MRSA accounted for more than 44% of post-cataract surgery cases [9]. This suggests that the incidence of MRSA endophthalmitis has increased significantly compared to 1.9% of total isolates that were MRSA in the Endophthalmitis Vitrectomy Study (EVS) [10]. However, current endophthalmitis prophylaxis measures using povidone-iodine surgical prep and chloramphenicol, are effective against MRSA [11,12].

The outcomes of MRSA endophthalmitis may also help to guide policy on pre-operative screening. Deramo et al. [8] found that 67% (total 4) of MRSA-isolates were associated with poor visual outcome [8]. Better outcomes were reported in the EVS [10], where 50% of *Staphylococcal aureus* cases had a visual acuity (VA) better than 20/100 (moderate visual loss or better outcome). However, a more recent case series in the USA demonstrated that 50% of MRSA cases had severe visual loss (VA worse than 20/400) [9].

In the survey, one of the units that reported a case of MRSA endophthalmitis decided not to screen for MRSA since the community acquired strain was more common in the locality. However, community acquired MRSA (CAMRSA) can cause soft tissue infections (lids and orbital) that are aggressive and spread rapidly [13]. Most strains also contain virulence factors such as Panton–Valentine leukocidin (PVL), a cytotoxin that destroys neutrophils and macrophages [14].

The final point to address is whether patients who are MRSA carriers (or high-risk) are a risk of transmitting MRSA to others. The reduction in the number of units that swabbed the eye for MRSA pre-operatively (31% vs 18%) may reflect that units may not be concerned about endophthalmitis but rather a risk of transmission to others. The DoH document on screening for MRSA colonisation briefly states this as a possibility [5]. In the cataract day-surgery setting, theoretically, the risk of transfer of carriage of MRSA between patients should be no higher than in outpatient clinics. The most relevant setting to cataract day-case surgery alluded to in the UK MRSA guidance is ambulance transportation. It recommends “most MRSA carriers may be transported with other patients in the same ambulance without any special precautions, other than changing the bedding used by the carrier” [4]. A history of in-patient stay is an independent risk factor for MRSA carriage [15]. However, a review of the literature revealed a relative paucity of information relating to outpatient visits, besides a retrospective cross-sectional survey which found that for patients in long-term care facilities, medical imaging was an independent risk factor for MRSA carriage, even when hospitalisation was accounted for [16]. It has also been found that there is a significant relationship between the length of hospital stay and the acquisition of MRSA [17]. Thus, the question remains: does an MRSA carrier represent a significant risk to others in a short-stay day surgery ward?

There are several resource implications if a decision is made to screen selected patients for MRSA carriage. At the pre-operative stage it would be necessary to identify these patients (through specific questions and to review notes and lab results), then to swab these patients and initiate treatment, if necessary. This would all represent an increased burden for the pre-assessment staff. Furthermore, the UK MRSA guidance suggests that swab sites should include groin/perineum [4] but this may not be practical in an eye clinic where a pre-assessment is being conducted. The DoH (England) document states that the primary site should be the nose, since it is the most common carriage site and, if a carrier is identified, a decolonisation regime should be started [5].

Ninety three (93)% of units that screened felt primary care were responsible for instituting decolonisation therapy, but guidance on whether this is appropriate for a day-case setting is again not clear. Decolonisation includes 5 days of antibacterial body shampoo and nasal cream and should be done irrespective of whether facilities are available to isolate the patient [5]. However, there are concerns that the use of nasal mupirocin antibiotic ointment may lead to resistant strains [18] and a review found that there is insufficient evidence to support use of topical antimicrobial therapy for eradicating nasal or extranasal colonisation with MRSA [19]. Nevertheless, the DoH document argues that decolonisation can significantly reduce the shedding of MRSA, and subsequently, the risk of infection and transmitting MRSA to others [5].

An alternative to decolonisation may be to set up an isolation area with barrier measures and to decontaminate the area, but it is believed that the most useful method to limit MRSA cross infection is careful hand hygiene between each episode of patient contact [20].

A limitation of our study is that not all units responded to our survey, nevertheless it does highlight a variation in screening practices. Contacting the pre-assessment nurses directly represented a reliable source of answering the questions posed. Although local infection control teams can represent an alternative source of information, pre-assessment nurses would know answers to questions, such as cases of MRSA endophthalmitis, due to discussion at regular departmental morbidity meetings which occur in NHS hospitals.

## Conclusions

This survey has highlighted the lack of clarity in current guidelines on whether day-case cataract surgery patients at high-risk of MRSA carriage should undergo screening. For a situation similar to the cataract day-case surgical setting, there is no strong evidence to suggest that MRSA carriage increases the risk of endophthalmitis or whether there is a significant risk of transmitting MRSA to others.

## Abbreviations

CAMRSA: Community acquired MRSA; DoH: Department of health; EVS: Endophthalmitis vitrectomy study; GP: General practitioner; HAIs: Hospital acquired infections; MRSA: Methicillin-resistant *staphylococcus aureus*; NHS: National health service; VA: Visual acuity.

## Competing interests

The authors declare that they have no competing interests.

## Authors’ contributions

LJ and LH drafted the manuscript. LH and SKW conceived the study and participated in the design. SKW conducted the first survey. LJ conducted the second survey. All authors read and approved the final manuscript.

## Pre-publication history

The pre-publication history for this paper can be accessed here:

http://www.biomedcentral.com/1471-2415/13/80/prepub
